# Symmetry-Defying Iron Pyrite (FeS_2_) Nanocrystals through Oriented Attachment

**DOI:** 10.1038/srep02092

**Published:** 2013-06-28

**Authors:** Maogang Gong, Alec Kirkeminde, Shenqiang Ren

**Affiliations:** 1 Department of Chemistry, University of Kansas, Lawrence, Kansas 66045, United States

## Abstract

Iron pyrite (fool's gold, FeS_2_) is a promising earth abundant and environmentally benign semiconductor material that shows promise as a strong and broad absorber for photovoltaics and high energy density cathode material for batteries. However, controlling FeS_2_ nanocrystal formation (composition, size, shape, stoichiometry, etc.) and defect mitigation still remains a challenge. These problems represent significant limitations in the ability to control electrical, optical and electrochemical properties to exploit pyrite's full potential for sustainable energy applications. Here, we report a symmetry-defying oriented attachment FeS_2_ nanocrystal growth by examining the nanostructure evolution and recrystallization to uncover how the shape, size and defects of FeS_2_ nanocrystals changes during growth. It is demonstrated that a well-controlled reaction temperature and annealing time results in polycrystal-to-monocrystal formation and defect annihilation, which correlates with the performance of photoresponse devices. This knowledge opens up a new tactic to address pyrite's known defect problems.

Iron pyrite (fool's gold, FeS_2_) is an eco-friendly material that is abundant in nature and is extremely promising for use as an active layer in photovoltaics, photoelectrochemical cells, broad spectral photodetectors and cathode material for batteries^1^,^2^,^3^,^4^. Pyrite boasts a strong light absorption (α > 10^5^ cm^−1^), a suitable band gap of E_g_ = 0.95 eV^5^. and an adequate minority carrier diffusion length (100–1000 nm)^6^,^7^, and more importantly, exhibits non-toxicity and near-infinite elemental abundance. Enhancing its excellent properties requires basic research on the controlled growth of pyrite, such as shape, size and stoichiometry. Controlled preparation of FeS_2_ nanocrystals with specific sizes and shape has been investigated in studies, involving the synthesis of zero dimensional (0D) nanoparticles^8^, one dimensional nanowires (1D)^9^, two dimensional (2D) thin hexagonal sheets^1^ and three dimensional (3D) nanocubes^10^,^11^,^12^,^13^. Synthesis-by-design and understanding underlying growth mechanisms is an especially important tool for targeted energy harvesting or storage applications. Therefore, tailoring the size, shape and properties of pyrite nanostructures is a major challenge that must be overcome before use in practical applications.

In the past, as classical crystal growth kinetics models, LaMer and Ostwald ripening (OR) theories have been widely used for the controlled synthesis of various colloidal nanoparticles, in which the initial nucleation and growth can be explained by the Gibbs-Thompson law^14^,^15^. More recently, a novel growth process called Oriented-Attachment (OA) has been identified which appears to be a unique mechanism during the development of nanoscale materials^16^,^17^,^18^,^19^,^20^,^21^. The aggregation controlled OA provides an important route by which nanocrystals grow, an explanation of how defects (dislocation) are formed and unique crystal morphologies, often symmetry-defying, can be produced. The OA process was first described by Penn and Banfield et. al^22^,^23^. Recently, Tang and Kotov reported the controllable synthesis of inorganic nanocrystal materials using the self-assembly based OA mechanism^24^,^25^. The interaction force among particles plays an important role in the OA process, such as dipole-dipole interaction, electrostatic repulsion, van der Waals interaction, and hydrophobic attraction^25^,^26^,^27^. The basics of the OA process are (1) primary nano-clusters or particles aggregate, (2) a rotational step to achieve collision of higher energy surfaces occurs, (3) removal of surfactants or absorbates, and finally (4) coherence is achieved by combination of the high surface energy facets into a single crystal that results in the reduction the overall surface energy of the particle. This coherence, while thermodynamically favorable, may also create line and plane defects and twining. These defects can lead to different properties of the material and give clues to crafting optimized FeS_2_ nanocrystals for device applications and can be used to explain poor performance of previous attempts at pyrite solar cell devices^2^.

In this study, a novel growth mechanism of FeS_2_ pyrite nanocrystals is presented. The new process exhibits a combination of LaMer theory for the initial quantum dot seeds followed by OA growth to create the shape, size and crystallinity of the FeS_2_ nanocrystals. The OA growth is observed in creation of four different shapes of FeS_2_ nanocrystals (cube, sheet, hexagonal plate and sphere) implying this is a dominant mechanism for FeS_2_ nanostructures. Observing an OA growth mechanism could offer insight into pyrite's known problems that have been attributed to vacancies and crystal defects that hold it back as a highly promising photovoltaic material^28^. High-resolution transmission electron microscopy (HRTEM) images show the progression from initial seeds to final monocrystal phase. To our knowledge, the OA growth has not been reported utilizing a hot-injection method, as usually a precipitation method is used to create the initial seeds and the final crystals. Finally, it is shown that FeS_2_ sheets created from the OA growth process can be integrated into a photodetector device and can be used as a probe for defect mitigation, and more importantly, shows the extent of recrystalization's effect on optoelectronic performance.

In the following report, evidence will first be presented for the OA growth in multi-shaped FeS_2_ nanocrystals and a proposed reasoning for final shape created in the nanocrystals. Characterization of FeS_2_ nanocrystals will be presented next followed by tuning of the nanocrystal size by utilizing OA kinetics. Finally, the performance of photodetector devices created out of FeS_2_ pyrite nanosheets will be presented and analyzed.

## Results

**Figure 1:**
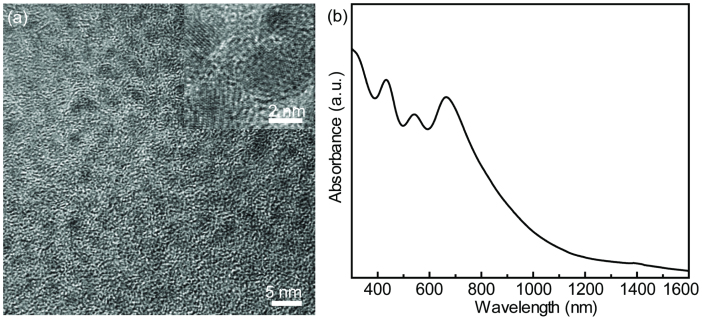
TEM images of FeS_2_ QDs (a), inset is the high resolution TEM image, and UV-Vis absorbance (b).

The initial step in synthesis of FeS_2_ nanocrystals consists of the creation of FeS_2_ quantum dot (QD) seeds. QD formations are realized by a rapid hot-injection of sulfur into an iron precursor solution, quickly creating QDs which show an average diameter of 2 nm with a narrow size distribution (Figure 1 and Supplementary Fig. S1) and create a transparent deep blue solution when dissolved in chloroform. Figure 1b shows the optical absorption spectrum of FeS_2_ QDs which exhibit strong quantum confinement and well-defined excitonic features, that match well with a previous report^29^. The OA process then proceeds utilizing the QDs as primary particle seeds. By controlling the injection temperature, different surface facet-rich nanocrystals can be obtained, which directs the collision or the attachment direction and thus control the cube or symmetry-defying sheet growth.

**Figure 2:**
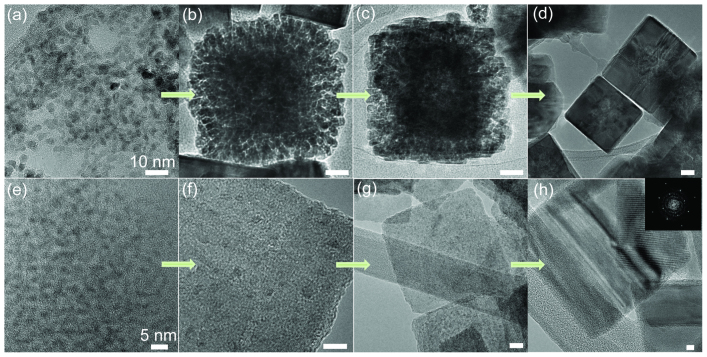
Sequences of TEM images show the detail of the attachment process. (a) FeS_2_ QD seeds; (b) seed collision; (c) seed coalescence; (d) recrystallization process from polycrystal to monocrystal. (e–h) FeS_2_ seeds evolved into single crystal nanosheet by coalescence and recrystallization process (inset of Fig. 2h shows Fast Fourier Transform of nanosheet).

Since different surface facets of FeS_2_ QD seeds exhibit different surface energy, anisotropic OA growth is realized by the combination of energetically unfavorable surface facets which will reduce the overall energy of the formed FeS_2_ nanocrystals. After the aggregation occurs (See Supplementary Fig. S2), the OA process continues with the formation of a polycrystalline structure followed by a recrystalization to a monocrystal. TEM images of each step for FeS_2_ nanocube formation are presented in Fig. 2a–2d. Optical absorption spectrum tracking structure changes are presented in Supplementary Fig. S3. Note that by the aggregation step, a cube-like shape can already be seen being formed (Fig. 2b). The OA growth is defined by the material's symmetry and the surface facets of FeS_2_ which exhibit the lowest energy^30^. By increasing the injection temperature from 393 K to 418 K, thin FeS_2_ pyrite {100} nanosheets are formed for the first time by the OA mechanism (Fig. 2e–2h). The small seeds can be seen within the sheet-like matrix (Fig. 2f), reminiscent of PbS sheets formed by OA growth^31^. In the case of the nanosheets, it is seen that final sheets grow thicker from aging (Fig. 2h). Supplementary Figure S4 presents the thickening evidence through the TEM cross section of the sheets at different growth time.

**Figure 3:**
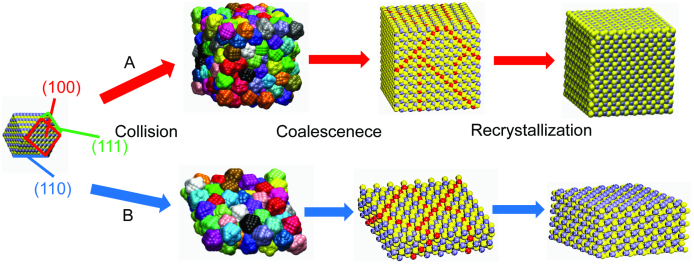
Schematic illustration of the cubic (pathway A) and sheet (pathway B) formation of FeS_2 _nanocrystals.

We interpret the symmetry-defying OA growth mechanism of our FeS_2_ nanocrystals based on the thermodynamic stability of different surface facets predicted by Barnard and Russo^30^. In their work, it is shown a truncated FeS_2_ nanocluster of 5 nm is made up with 6 {100}, 8 {111} and 12 {110} surface facets. Figure 3 presents a depiction of these nanoclusters and the paths to different shape formation seen in this study. In the case of cube growth (path A), a relatively larger FeS_2_ QD seed is created at a lower injection temperature, which results in mainly {100} surface planes being formed. The FeS_2_ QD seeds are stabilized by the OA preferentially along {100} facets to form cubic FeS_2_ nanocrystals with {100} surface planes. Regarding the FeS_2_ nanosheet formation, creation of relatively smaller crystallites with higher {110} surface area explains the in-plane attachment. FeS_2 _QD seeds with {110}-rich surfaces are created when the temperature of the injection is increased. We interpret the thin FeS_2_ nanosheet formation by the aggregation of the seeds through the {110} surface plane, shown in Fig. 3, pathway B. Since {110} surface facets of FeS_2 _seeds have higher surface energy, they are preferentially consumed by the in-plane 2D attachment, resulting in the FeS_2 _nanosheet formation. Conversion to the thicker sheet structures most likely occurs through attachment of the sheets prevalent {100} surface, as the planar dimension does not change in size, shown in Supplementary Fig. S4. The ability to control initial seeds and their surface facets will prove valuable in extracting the obtainable shapes of FeS_2_ nanocrystals (see Supplementary Fig. S5), which have shown vastly different properties in optoelectronic and electrochemical devices by us (this will be discussed in a future report).

**Figure 4:**
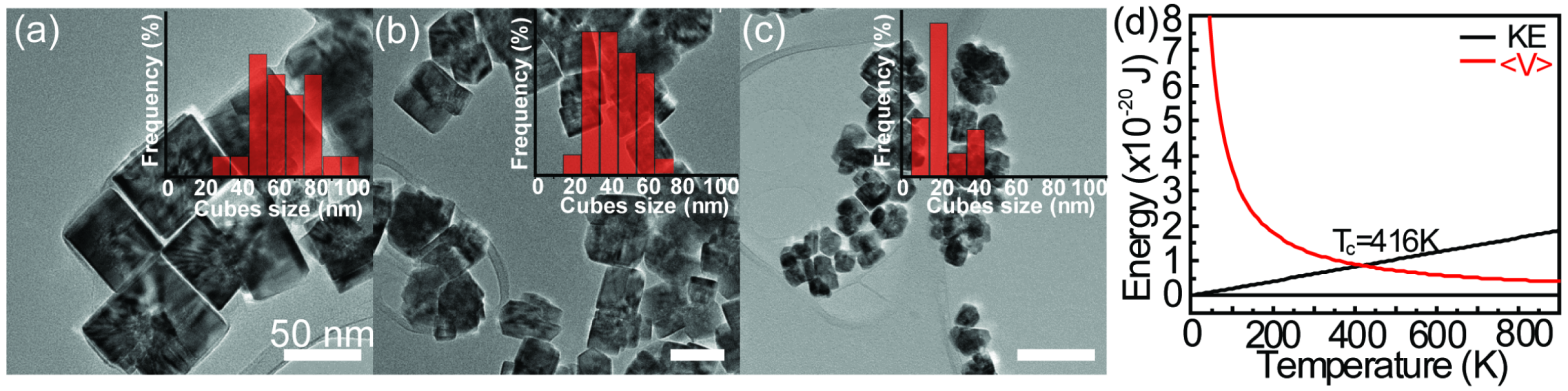
FeS_2_ cubic nanocrystals at different growth temperature. (a) FeS_2_ nanocube at 493 K growth, (b) FeS_2_ nanocube at 523 K growth and (c) FeS_2_ nanocube at 546 K growth. (d) The kinetic energy (KE) and the dipole-dipole potentials <V> as the function of reaction temperature for FeS_2_ nanocrystal growth.

It has been observed and explained kinetically that in the OA growth model, higher growth temperature leads to smaller particles due to the extra energy allowing for easier de-adsorption of the particles during the collision step of the OA based growth^32^. The OA controlled tunability of FeS_2_ nano-crystal dimensions is confirmed by varying growth temperature of the cubic synthetic route. Figure 4a–4c shows TEM images of FeS_2_ nanocrystals when the growth temperature was 493, 523 and 543 K, respectively. Quantitatively, it is seen that as the growth temperature increases, the average size of the final FeS_2_ nanocrystal decreases from 64 nm, 43 nm, and 23 nm, respectively, providing additional evidence of an OA controlled growth mechanism. Another key difference between OR and OA growth in nanocrystals is particle size dependence on the growth time. In OR, as stated above, bigger particles grow at the expense of smaller particles, making size increase as time progresses. In OA, the particles attach to create a more stable particle, and then usually cease to grow afterwards (there exists cases where after an OA step, OR takes over and some growth still occurs). This leads to a stagnation of size after the OA growth has taken place. In this study, there exists a point where the FeS_2_ nanostructures stop growing in size. Supplementary Figure S6 shows the size of cubic structures at 40 min and 120 min into the synthesis and there is no observed change in the overall sizes. Controlling the size of FeS_2_ nanoparticles is an important goal, as it has been stated that only a 40 nm film is required in devices due to the material outstanding absorption coefficient^12^.

The existence of different size and shape of FeS_2_ nanocrystals suggests different collision and coalescence behavior of FeS_2_ seed crystallites. In the OA growth process, the reaction temperature dominates the collision and the coalescence which is attributed to the particle's medium- and short-range interactions, such as Van der Waals forces and dipole-dipole interaction forces. Van der Waals forces are estimated to be less than 0.5 RT, which is not enough to stabilize superstructures under ambient conditions^33^. The force capable of producing FeS_2_ polycrystals is thus believed to be the long range dipole-dipole attraction. The energy of dipole attraction between FeS_2_ QD seeds can be calculated using the classical formula <V> = −μ^2^/2πε_o_*r*(*r*^2^ − *d*_NP_^2^). Estimating the center-to-center interdipolar separation *r* to be 2.6 nm, the FeS_2_ QD seed diameter to be *d*_NP_ = 2 nm and taking the dipole moment for this size μ = 17.6D, the energy of dipole attraction <V> is equal to 5.2 kJ/mole^34^. In the weakly flocculated colloidal state, the dipole-dipole potential can also be expressed as a function of temperature T. When dipole-dipole potentials <V> and kinetic energy (KE) are plotted as a function of T (Fig. 4 (d)), an intersection represents a critical temperature, T_c_ of the system at 416 K. T_c_ represents when the thermal energy exceeds the attractive potential energy among FeS_2_ seeds. If the reaction temperature is lower than the T_c_, the attractive dipole-dipole potential energy dominates the OA process by coalescence. Once the reaction temperature exceeds T_c_, the KE will control the OA growth, which is dictated by the collision. The size of FeS_2_ nanocrystals will be controlled by reaction time in the coalescence state. In our FeS_2_ synthesis, we control the coalescence and collision to yield FeS_2_ nanocubes with different sizes, by tuning the heating rate and thus the reaction time within the coalescence state (Fig. 4a–4c).

**Figure 5:**
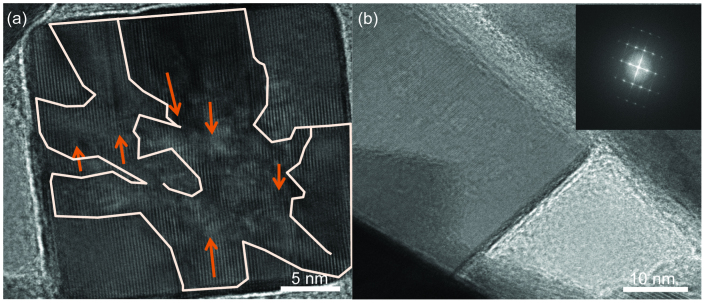
HRTEM images of one cubic FeS_2_ nanocrystal recrystallization process. (a) A polycrystal FeS_2_ nanocube at 40 min into synthesis. Different domains are separated by stacking faults (outlined as arrows) due to the collision of the OA growth. (b) A monocrystal FeS_2_ nanocube after aging 120 min and the inset shows the {100} diffraction pattern.

While the shape and size control is an important goal in the FeS_2_ system, defect mitigation may be the most crucial aspect in achieving optimal FeS_2_ nanostructures. It has been widely accepted that the defects (such as, surface states, dislocations, twins, etc) of FeS_2_ nanocrystals dictate their optoelectronic and electrochemical applications, therefore a strategy to achieve high quality crystalline FeS_2_ needs to be identified. Polycrystalline-to-monocrystalline conversion of the OA growth can be utilized to create highly crystalline FeS_2_ nanocrystals. Figure 5a shows a HRTEM image of a FeS_2_ nanocube at 40 min into the synthesis. It can be seen that different domains (outlined by lines) exist, while stacking faults can clearly be seen (highlighted by arrows) due to the collision of the OA growth process. These defects are detrimental to material quality as they act as the charge recombination centers for excitons and need to be eliminated to create optimal solar cell devices. Upon greater lengths of aging time in the same pyrite solution, it is seen that these defects are eventually eliminated. Figure 5b shows a monocrystalline cubic FeS_2_ nanocrystal aged for 120 min and the inset shows [100] growth diffraction pattern. This suggests that longer aging times will be beneficial for FeS_2_ nanomaterial, due to the stagnation of the OA controlled FeS_2_ growth, the longer aging times should not interfere with shape/size.

## Discussion

**Figure 6:**
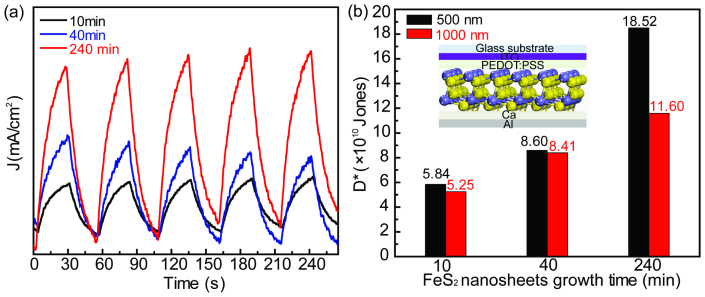
The FeS_2_ nanosheet photodetector performance. (a) The reproducible on/off switching of the device upon AM-1.5 sun light at a bias of 1.0 V. (b) Detectivity under 500 nm and 1000 nm light illumination, dependent on the growth time of FeS_2_ sheets (inset shows the schematic of FeS_2_ sheets device).

As stated above, it is widely agreed that defects in pyrite material is the limiting factor for performance of devices^2^,^35^,^36^,^37^. In this study, it is seen that the pyrite particles eventually reach a maximum size, and then begin to convert from poly-crystalline to mono-crystalline, which will reduce the defects that are caused by the OA mechanism. To test this hypothesis, a series of photodetector devices were fabricated, using FeS_2_ nanocrystals with varying aging times to examine the effect of crystallinity on the device performance. The time-dependent photoresponse of FeS_2_ nanosheets are shown in Fig. 6 (a). The current difference between irradiation (light on) and dark (light off) is clearly enhanced by increasing the aging time of the FeS_2_ nanosheets. The figure of merit we use to compare photodetector performance is the normalized detectivity (D*)^38^. D* values of the FeS_2_ nanosheet devices are 5.84 × 10^10^, 8.60 × 10^10^, and 1.85 × 10^11^ Jones, corresponding to 10 min, 40 min, and 240 min aging time of nanosheets, respectively (shown in Fig. 6(b)). Since FeS_2_ nanosheets demonstrate a strong absorbance in the near infrared (NIR) wavelength, they could work as the NIR photodetector. Figure 6b shows the performance under 1000 nm illumination, which confirms excellent NIR performance and again demonstrates the effect of crystallinity on the photodetector performance. The R_λ_ and D* of FeS_2_ devices at 1000 nm illumination show 0.16 A/w and 5.25 × 10^10^ Jones (10 min aging), 0.60 A/w and 8.41 × 10^10^ Jones (40 min aging) and 3.94 A/w and 1.16 × 10^11^ Jones (240 min aging). The enhanced detectivity can be attributed to the increased crystallinity as a result of increased aging time during the FeS_2_ growth. These results support that defects within the material are being mitigated due to the recrystallization of the FeS_2_ nanomaterials. More detailed photodetector device studies are underway to utilize their unique IR absorbance.

A symmetry-defying OA growth and its implications for different shaped FeS_2_ nanocrystals have been presented and discussed. FeS_2_ nanocrystals show the growth starting with FeS_2 _QD seeds, which exhibit excitonic absorption behavior and enable further OA growth for shape and size control. A growth pathway model and thermodynamic reasoning are then presented to facilitate understanding of shape and size control in the FeS_2_ system. Shape and crystallinity of FeS_2_ nanocrystals is shown to be dependent on reaction temperature and aging time. Photodetector performance is shown to be correlated with crystallinity, offering support for defect mitigation in the material. Observation of the symmetry-defying OA growth in FeS_2_ nanocrystals and its effect on crystallinity will facilitate FeS_2_ along on its path to becoming a “golden” material for sustainable energy applications. Controlling crystallinity is a key point in the generation of complex functional nanomaterials. Self-assembly of particles into larger single-crystalline objects by the OA mechanism, is one of the most promising approaches in nanotechnology. This OA evolution process can be adjusted by cosolvents^26^,^31^, high pH value^34^, temperature and time^24^. A well-controlled reaction conditions in the OA process can facilitate the high quality nanocrystal growth.

## Method

### Synthesis

The FeS_2_ nanocube synthesis starts with 0.5 mmol FeCl_2_ in octadecylamine (ODA, 12 g) loaded into a three neck flask and degassed and back filled with argon, heated to 393 K and allowed to decompose for 120 min. Another three neck flask is then loaded with 4 mmol sulfur powder in diphenyl ether (5 mL), is degassed and back filled with argon, and heated to 343 K for 1 hour to dissolve. The sulfur solution is then quickly injected into the Fe-ODA precursor at the temperature of 393 K. After injection, the combined solution was heated to 493 K and aliquots at different time intervals were taken for UV-vis-NIR absorption test and HRTEM characterization. For FeS_2_ thin sheets, injection temperature of the Fe-ODA precursor is raised to 418 K with everything else kept the same. Particles were separated by centrifugation and purified by being re-dissolved and crashed in chloroform-methanol. The final particles were dispersed in chloroform for storage and characterization.

### Materials Characterization and Devices fabrication

All UV-Vis-NIR absorbance spectra were obtained on a UV-3600 Shimadzu Spectrophotometer. HRTEM images were obtained using Field Emission FEI Tecnai F20 XT. The photodetector devices are fabricated as following: A PEDOT:PSS layer is used to flatten the ITO patterned glass substrate and serves as a hole transporting layer. The FeS_2_ nanosheets were dissolved in chloroform with a concentration of 25 mg/mL. The FeS_2_ nanosheets were deposited on the PEDOT:PSS surface by spin coating method at the speed of 1500 RPM, Then, a thin layer of calcium (~10 nm) was thermally evaporated. Finally, a patterned aluminum electrode (~80 nm) was evaporated on the top surface of the calcium, completing the device.

## Author Contributions

M.G. and A.K. carried out the synthesis, characterization and analyzed the data, and wrote the paper. The authors M.G. and A.K. contributed equally to this work. S.R. supervised the project and conceived the idea and experiments. All the authors discussed the results, commented on and revised the manuscript.

## Supplementary Material

Supplementary Information Supporting Information
