# Supplementary material for: Symmetry-Defying Iron Pyrite (FeS2) Nanocrystals through Oriented Attachment
Source: Sci Rep. 2013 Jun 28;3:2092. doi: 10.1038/srep02092 (PMC3697776; doi:10.1038/srep02092)
Supplement: Supplementary Information — Supporting Information [file srep02092-s1.pdf]

Supplementary Information

Symmetry-Defying Iron Pyrite (FeS_2) Nanocrystals through Oriented Attachment

Maogang Gong, Alec Kirkeminde, Shenqiang Ren*

Department of Chemistry, University of Kansas, Lawrence, Kansas 66045, United States.

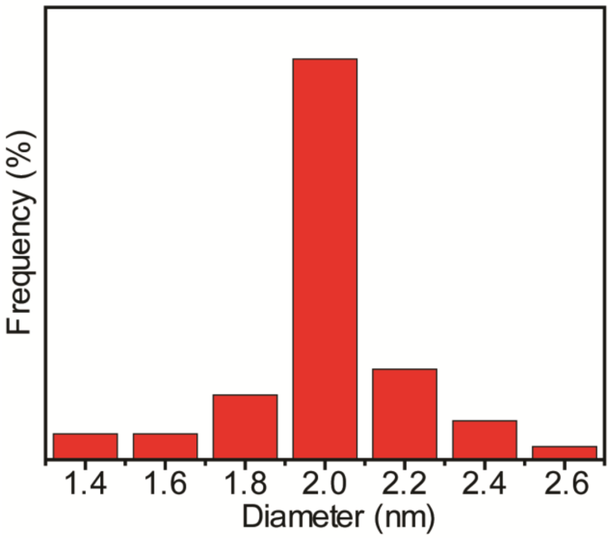

Figure S1: FeS_2 QDs diameter statistics histogram.

Figure S1 shows the FeS_2 QDs diameter distribution with an average of 2 nm. Figure S2 presents FeS_2 collision and coalescence. As the size of FeS_2 nanocrystals increase, the collision and coalescence could occur between any of two multilevel nanocrystals.[1] Meanwhile, the collision cross-section of FeS_2 nanocrystals enlarges and the velocity decreases. Putting two factors together, a rapid decrease of the growth rate is observed until the process ends. It can be seen that each bigger nanocrystal is typically formed by several smaller seeds attaching together via a common crystallographic orientation (Figure S2(a)). Figure S2 (b, c) illustrate the grain edges of coalescence. Thus, most formed FeS_2 nanocrystals have irregular shapes and abrupt edges, exhibiting the particular character of crystal growth by the OA mechanism.

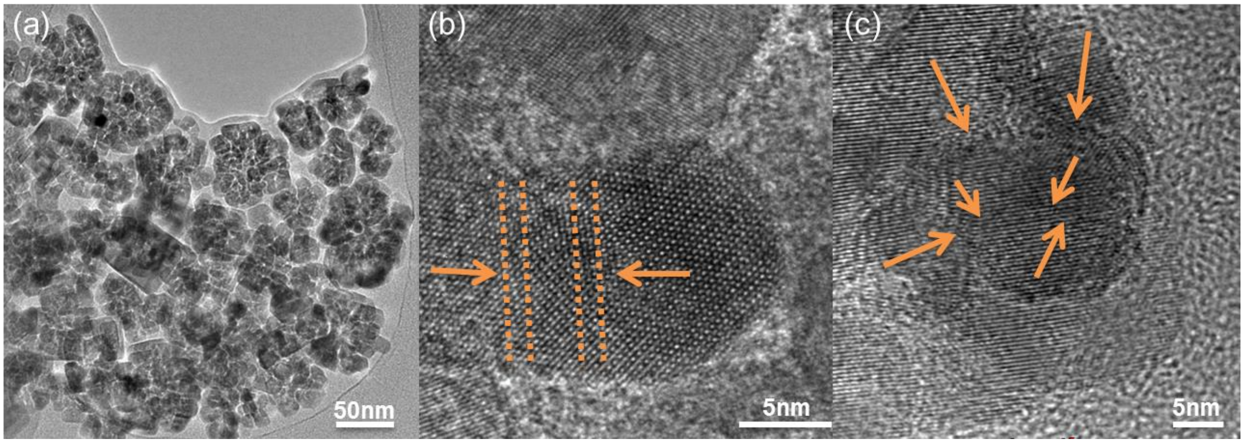

Figure S2. TEM images FeS_2 collision and coalescence. (a) The collision and coalescence of small FeS_2 seeds, (b, c) HRTEM images of FeS_2 aggregates (the arrows indicate typical features of the OA process).

Figure S3 presents UV-Vis absorption spectrum of particles as the reaction progresses. Excitonic peaks disappear 30 min into the reaction and as time goes on the absorption peak shifts farther into the near IR.

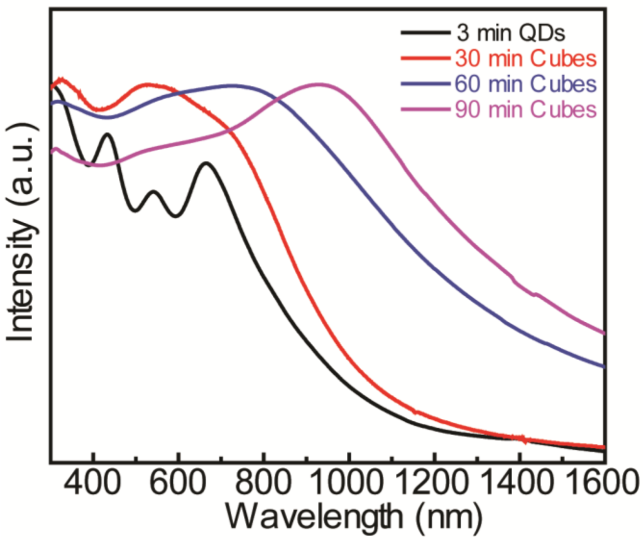

Figure S3. UV-Vis absorption spectrum of FeS_2 particles depending on reaction time.

Figure S4 illustrates the FeS_2 square nanosheet structures. The FeS_2 ultrathin nanosheet forms after 10 min reaction, as shown in Fig. S4 (a, b). Upon further reaction time (240 min), the planar geometry and size of FeS_2 nanosheet does not change, but it grows thicker along the thickness direction (Figure S4 (c, d)). The FeS_2 nanosheet growth process is also controlled by the OA mechanism and the thinner nanosheet continuous collide and attach through the {100} surface, then coalescence together to form thicker nanosheet. When the reaction time reaches 360 min, the FeS_2 nanosheet evolved into a nanocube structure (Figure S4 (e, f)).

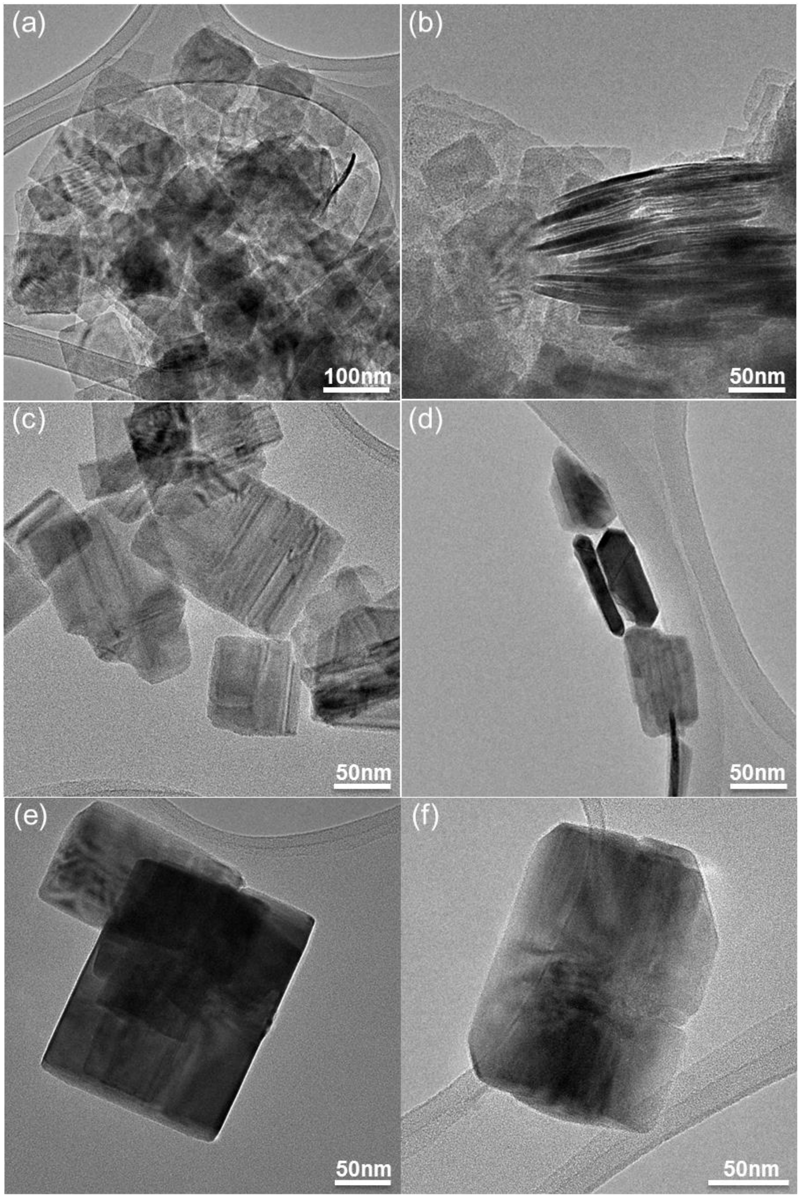

Figure S4. TEM images of FeS_2 square nanosheet structures. (a) ultrathin FeS_2 nanosheet and (b) cross-section image of FeS_2 nanosheet after 10 min, (c) nanosheet and (d) cross-section images after 240 min, (e) FeS_2 nanocube structure and (f) cross-section images after 360 min.

Figure S5 shows the symmetry-defying FeS_2 hexagonal plate structure growth process. For preparing FeS_2 hexagonal crystals, $Fe(CO)_5$ used as the iron source and same molar amount as the standard $FeCl_2$ synthesis (220°C injection). The FeS_2 hexagonal plate growth process can be divided into two stages. After inject the $Fe(CO)_5$, small nanoplates are formed by nucleation process shown in Fig. S5 (a). The edge lengths of these nanoplates vary from 5-15 nm, and the shape is irregular hexagon. The second stage, small nanoplates grow into bigger symmetric hexagon mircoplates by the collision and coalescence (Figure S5 (b)). Figure S5 (c) shows that the microplates are polycrystalline and made up of the initial nanoplates. Upon increasing the reaction time, the polycrystal plates evolved into single crystal by

recrystallization under thermal conditions (Figure S5 (d)). The FeS_2 hexagonal plate growth mechanism has been reported in our previous work.[2]

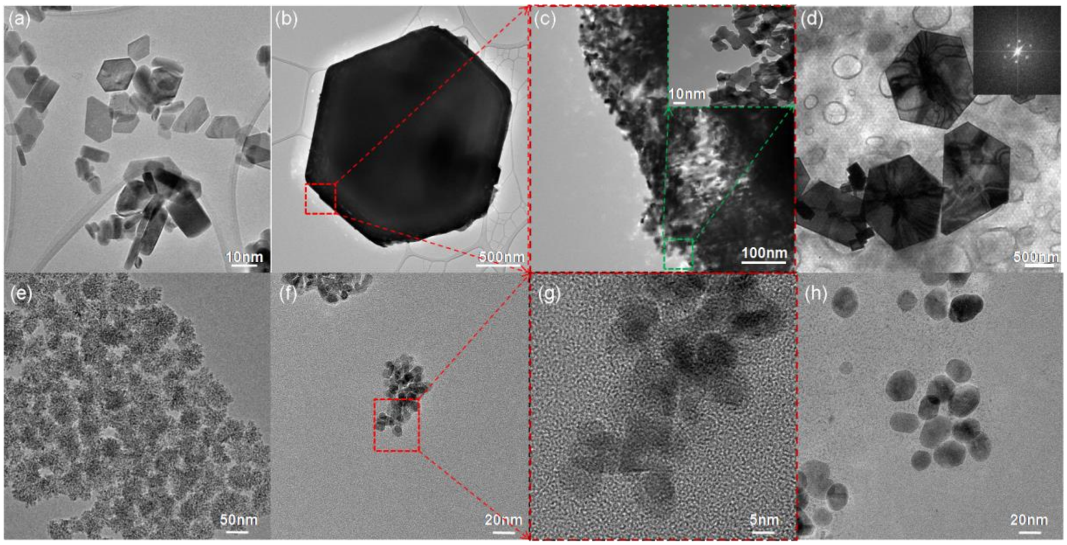

Figure S5. FeS_2 hexagonal plate and nano-sphere structure growth process. (a) FeS_2 nanoplates, (b) FeS_2 polycrystal microplates, (c) magnified image of polycrystal microplates (the inset shows magnified region highlighted in the green region), (d) single crystal FeS_2 hexagonal microplate (the inset shows the Fast Fourier Transform of microplate), (e-h) FeS_2 nano-sphere formation via the OA growth.

FeS_2 cubes grown at different times are presented in Figure S6. Comparing Figure S6 (a) (40min) and Figure S6 (b) (120 min), the size of FeS_2 cubes show no apparent difference, which indicates that the late stages of growth consisted of a recrystallization process and no coalescence occurs due to the larger particles velocity decrease. If we assume FeS_2 particles dispersed in the ODA solution with a high degree of freedom for rotation and translation motion, the oriented attachment in the dispersed colloidal state can be quantitatively described by the Ribeiro's model[3], and the kinetics equation can be expressed as:

$$r_{eq}^3 - r_i^3 = \frac{\frac{Nk_BT}{\eta}[\mathrm{A}]_0 t}{1+\frac{Nk_BT}{\eta}[\mathrm{A}]_0 t} \cdot r_i^3$$

Where r_i is the initial mean particle radius, r_{eq} is the equivalent radius at a reaction time t, N is the total number of particles, k_B is the Boltzmann constant, T is the absolute temperature, $[\mathrm{A}]_o$ is the initial concentration of particles (particles/volume), η is the liquid-medium viscosity, and t is reaction time. Since all terms are constant, except t, the above equation behaves as a function of $f(x)=x/(1+x)$. Based on above analysis, the OA kinetic growth process of FeS_2 nanocrystals can be qualitatively expressed as Figure S6(c). After a long period of reaction time, the FeS_2 particle size stabilizes and reaches a constant value.

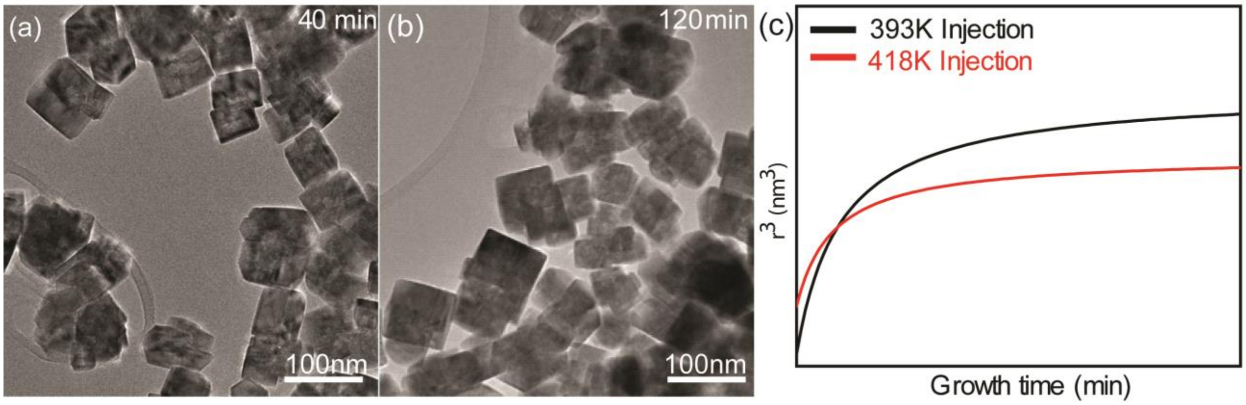

Figure S6. FeS_2 cubes grown at (a) 40 min and (b) 120 min. No size change is observed. (c) OA growth kinetic curves.

Figure S7 shows the TEM images of amorphous structure FeS_2 at 392 K reaction temperature.

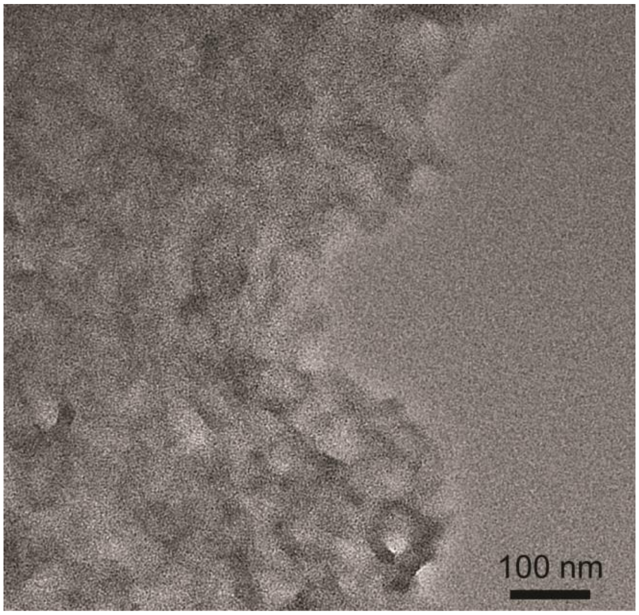

Figure S7. TEM image of FeS_2 amorphous structure.

1 Zhang, J., et al., A multistep oriented attachment kinetics: Coarsening of ZnS nanoparticle in concentrated NaOH, *J. Am. Chem. Soc.* **128,** 12981-12987 (2006).

2 Kirkeminde, A. and Ren, S. Q. Thermodynamic control of iron pyrite nanocrystal synthesis with high photoactivity and stability, *J. Mater. Chem. A.* **1**,49-54 (2013).

3 Ribeiro, C., Lee, E. J. H., Longo, E. & Leite E. R. A kinetic model to describe nanocrystal growth by the oriented attachment mechanism, *Chemphyschem*, **6**, 690-696 (2005)
